# Fabrication and Characterization of Solid Composite Yarns from Carbon Nanotubes and Poly(dicyclopentadiene)

**DOI:** 10.3390/nano10040717

**Published:** 2020-04-10

**Authors:** Wenbo Xin, Joseph Severino, Arie Venkert, Hang Yu, Daniel Knorr, Jenn-Ming Yang, Larry Carlson, Robert Hicks, Igor De Rosa

**Affiliations:** 1 Materials Science and Engineering, University of California Los Angeles, 410 Westwood Plaza, Los Angeles, CA 90095, USA; joseph.severino@hotmail.com (J.S.); jyang@seas.ucla.edu (J.-M.Y.); 2 Chemistry Department, Nuclear Research Center Negev (NRCN), 84190 Beer Sheva, Israel; venkert@gmail.com; 3 Chemical & Biomolecular Engineering, University of California Los Angeles, 420 Westwood Plaza, Los Angeles, CA 90095, USA; yuhang0485@gmail.com (H.Y.); hicksmaterials@gmail.com (R.H.); 4 Combat Capabilities and Development Command, Army Research Laboratory, 6300 Rodman Road, Aberdeen Proving Ground, MD 21005, USA; daniel.b.knorr.civ@mail.mil; 5 Institute for Technology Advancement, University of California Los Angeles, 410 Westwood Plaza, Los Angeles, CA 90095, USA; lcarlson@ita.ucla.edu

**Keywords:** carbon nanotube, composite yarns, dicyclopentadiene, consolidation and alignment, strengthening mechanism

## Abstract

In this report, networks of carbon nanotubes (CNTs) are transformed into composite yarns by infusion, mechanical consolidation and polymerization of dicyclopentadiene (DCPD). The microstructures of the CNT yarn and its composite are characterized by scanning electron microscopy (SEM), high resolution transmission electron microscopy (HRTEM), and a focused ion beam used for cross-sectioning. Pristine yarns have tensile strength, modulus and elongation at failure of 0.8 GPa, 14 GPa and 14.0%, respectively. In the composite yarn, these values are significantly enhanced to 1.2 GPa, 68 GPa and 3.4%, respectively. Owing to the consolidation and alignment improvement, its electrical conductivity was increased from 1.0 × 10^5^ S/m (raw yarn) to 5.0 × 10^5^ S/m and 5.3 × 10^5^ S/m for twisted yarn and composite yarn, respectively. The strengthening mechanism is attributed to the binding of the DCPD polymer, which acts as a capstan and increases frictional forces within the nanotube bundles, making it more difficult to pull them apart.

## 1. Introduction

Carbon nanotubes (CNTs) have unique structures and properties that could be advantageous in forming composites for structural applications [1,2], flexible sensing substrates [3], and multi-functional devices [4]. The ultimate tensile strength (UTS) of a carbon nanotube has been measured to be 150 GPa [5]. Typical multi-walled carbon nanotubes and bundles have strengths between 20 and 80 GPa [6], Young’s moduli between 0.9 and 1.2 TPa, and elongations to failure between 5% and 15% [7]. However, the utilization of these properties is a challenge, because van der Waals forces are the only means of transferring load between individual CNTs and the macroscopic structure. In the case of continuous CNT assemblies, the individual nanotubes agglomerate into larger bundles [8]. Methods of spinning from CNT solutions, arrays and aerogels can transform these bundles into a loose network that extends three dimensionally into macroscopic assemblies, such as sheets, yarns, ribbons and foams [9,10,11].

Fibrous assemblies of carbon nanotubes are originally prepared from dispersed solutions that are injected with a syringe pump into a moving coagulation bath [9]. The resulting filaments demonstrate a very low UTS, which are in the range of 50 to 150 MPa. Researchers have devoted tremendous efforts to enhancing the mechanical and electrical properties of CNT assembled bundles, yarns and sheets. Jiang et al. [10] found a more direct fabrication route by growing aligned arrays of carbon nanotubes that form fibers or films when the CNTs are drawn from the array. Zhang et al. [12] employed a twisting process, which stabilized and consolidated the structure, to produce nanotube yarns with an UTS over 480 MPa. The mechanical properties are boosted by consolidation, because the contact area between the nanotube bundles increases, thereby enhancing the load transfer. Consolidation of CNT assemblies is often achieved through mechanical treatments, such as stretching [13], twisting [14], rolling [15] and wire die drawing [16], but it can also be obtained by evaporating solvents to generate surface tension between the bundles [17,18]. A combination of these treatments can lead to CNT assemblies with improved tensile strengths of 3.0–5.5 GPa [19] for meter-long lengths and up to 9.6 GPa for short samples [15] with the electrical conductivity up to 8.5 × 10^6^ S/m [20].

It is well known that polymer incorporation can improve the mechanical properties of CNT assemblies by connecting non-adjacent CNTs and increasing load sharing between nanotubes [21]. Dispersion-based fibers that incorporated polyvinyl alcohol (PVA) to form a composite showed significantly increased toughness [22]. Ma et al. developed a process to incorporate epoxy and PVA resins into carbon nanotube yarns. After consolidation by twisting, the tensile strength was approximately 500 MPa without resin and 1.2 GPa for both infused with epoxy and PVA [23]. Furthermore, researchers have investigated the strengthening effects of a variety of other thermosetting and thermoplastic polymers on CNT assemblies, including polyimide [24], bismaleimide [24,25], polyethylenimine [26], polydopamine [27] and so forth. These polymer infiltrations can effectively increase the tensile strength of CNT assemblies up to 4.04 GPa [27].

Dicyclopentadiene (DCPD) is a promising candidate for infusion into CNT materials, owing to unique characteristics such as low viscosity (~10 cPs), low density (~1 g/cm^3^) [28], high toughness when compared to other thermosetting polymers [29], remarkable high velocity impact resistance [30] and a high cross-link density [31] for improved load transfer. The structure of DCPD consists of a cyclopentene moiety and a strained norbornene moiety with one shared edge, and it can be cured with Grubbs catalyst via ring-opening metathesis polymerization [32]. Because it is a monomeric resin, infusion is not inhibited by chain length. One of the pioneering works forming a nanotube composite based on DCPD resin was done by Jeong and Kessler [33]. The samples contained up to 0.4 wt% of dispersed and functionalized CNT powders in DCPD polymer. Compared with the pristine resin, CNT addition increased the energy to failure by over nine times, and failure changed from local crack propagation to ductile necking. However, there have been relatively few reports on the utilization of DCPD polymer in CNT assemblies (yarns or sheets) for improving the mechanical and electrical properties.

Herein, we demonstrate the fabrication of high-volume fiber fraction, consolidated, nanocomposites by infusion and polymerization of DCPD polymer within carbon nanotube yarns. To the best of our knowledge, this is the first investigation showing DCPD-infused CNT assemblies. We effectively improved both the mechanical and electrical properties of CNT yarns using this strategy. The microstructures of the carbon materials before and after incorporation of the resin are fully characterized by electron microscopies. A plausible capstan strengthening mechanism is proposed based on the experimental results.

## 2. Materials and Methods

### 2.1. Materials

Carbon nanotube ribbons and sheets were obtained from Nanocomp Technologies Inc. (Merrimack, NH, USA). The growth furnace and feedstock formulation were similar for both the sheets and ribbons, and therefore, the CNT in the two material forms were chemically indistinguishable. The ribbons and sheets differed only in the collection process that formed them into their material format [34]. As a result, they were used interchangeably to analyze structure and chemistry. The ribbons were 4 tex (g/km) with a skeletal density of 1.24 ± 0.30 g/cm^3^. The density of the sample was measured using a Micromeritics Accupyc 1330 pycnometer (Micromeritics Instruments Corp., GA, USA) employing He gas that was calibrated within three days of the measurement. The reported density values are an average of 15 measurements, reported with the standard deviation. The sheet areal density was 15 g/m^2^ and the resin used was primarily dicyclopentadiene, with a lesser amount of other constituents. For room temperature processing, 24 wt% tricyclopentadiene (CAS# 36806-65-2) was added to the resin. This reduced the melting point below 32.5 °C [31]. The polymerization reaction was catalyzed by a second generation Grubbs catalyst with molecular weight 792.87 au. Crystalline catalyst powder (96 µg) was suspended in 2 g of mineral oil that was, in turn, used to polymerize 100 g of DCPD resin.

### 2.2. Yarn Fabrication

To form CNT yarns without resin, ribbons were grasped by the ends in a twisting fixture. Initially, 0.25 N tension was applied to impart strain alignment. Then, a number of turns were introduced until approximately 1 helical revolution per millimeter was achieved. The tension was then increased to 0.5 N, where untwisted portions remaining from the previous step were aligned. A final twist was then introduced to achieve approximately 2 revolutions per millimeter. Composite yarns were fabricated by placing nanotube ribbons into catalyzed resin, mixed at 1-part catalyst to 50 parts resin by weight, and soaking for 5 min to allow time for infusion. Then the wet ribbon was removed from the DCPD bath and application of stretch and twist was done in a manner similar to the CNT yarn described earlier. The tension was maintained, and once the resin formed a gel, the yarn was placed in an oven at 120 °C for 3 h to form a cured nanocomposite. The yarn samples were then segmented into individual specimens for mechanical testing and mounted to paper frames with a 10 mm gage length.

### 2.3. Characterization

The mechanical properties were measured in tension using an Instron 5966 universal test system (Instron, Norwood, MA, USA) with a displacement resolution better than 0.1 µm. Samples were affixed into custom grips and the paper frames cut just prior to testing. Loading was performed at constant displacement rate of 5 mm/min with a 50 N load cell. A conversion from linear density [35] was used to calculate tensile stress, according to Equation (1),

(1) $$\sigma_{T}=A\frac{P_{load}}{\lambda }\times \rho_{cnt}$$

where $\sigma_{T}$ is the tensile stress (N/m^2^), $P_{load}$ is the measured load (N), *λ* is the linear density (tex = g/km), $\rho_{cnt}$ is the skeletal density of nanotubes (1.24 g/cm^3^) and *A* adjusts units with a value of 10^9^ cm^3^/km·m^2^. This approach normalizes to the skeletal stress carried by the carbon nanotube network. The linear density of the dry CNT yarn was also used for normalizing the stress in cured and uncured composites. This was done to allow comparison of the stress carried by carbon nanotubes in the three sample arrangements.

The nanocomposite structure was characterized by high resolution scanning electron microscopy (SEM). Two systems were utilized: a FEI Nova NanoSEM 230 (FEI company, Hillsboro, OR, USA) with 1.0 nm resolution in immersion mode, and FEI Nova 600 dual beam HRSEM (FEI company, Hillsboro, OR, USA) with focused ion beam (FIB) which was used for cross-sectioning. For sub-nanometer characterization, a FEI Titan 80–300 keV S/transmission electron microscopy (TEM) system (FEI company, Hillsboro, OR, USA) was utilized with 80 keV accelerating voltage. Raman spectra are acquired from the CNT sheet using a Renishaw In-Via Raman spectrometer (Renishaw, Wotton-under-Edge, UK). The laser wavelength and power are 785 nm and 0.25 mW, respectively, at an objective lens magnification of 50× and a grating spacing of 1200 L/mm. Spectral resolution in our data is 1 cm^−1^. Finally, the thermogravimetric analysis (TGA) of CNT and CNT composite was performed on DSC/TGA system (SDT650, TA instrument, New Castle, DE, USA) in a mixed gas (He/O_2_ = 80/20) at the flow rate of 100 mL/min. The heating rate was 20 °C/min.

## 3. Results

### 3.1. CNT Assemblies

**Figure 1:**
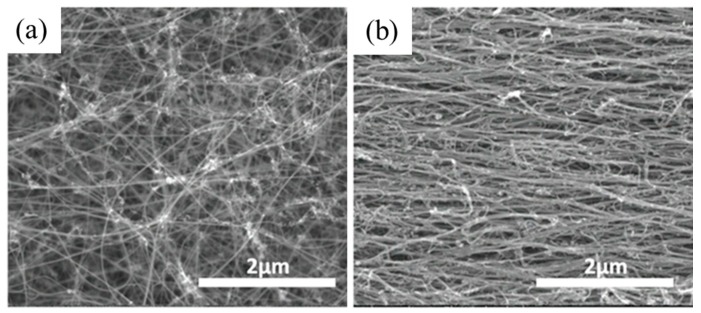
Carbon nanotube sheets before (**a**) and after (**b**) elongation to 30% strain.

**Figure 2:**
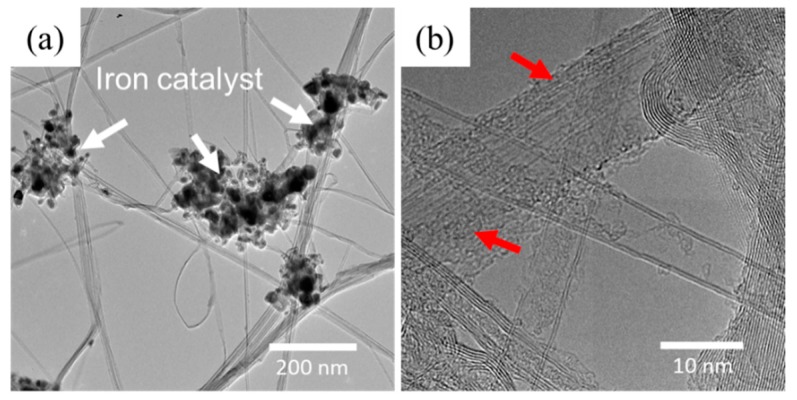
Transmission electron microscopy (TEM) characterization of carbon nanotubes (CNT). (**a**) Low magnitude image showing agglomeration of iron catalyst nanoparticles (highlighted by white arrows). (**b**) high magnitude image showing individual nanotubes covered by amorphous carbon (red arrows).

**Figure 3:**
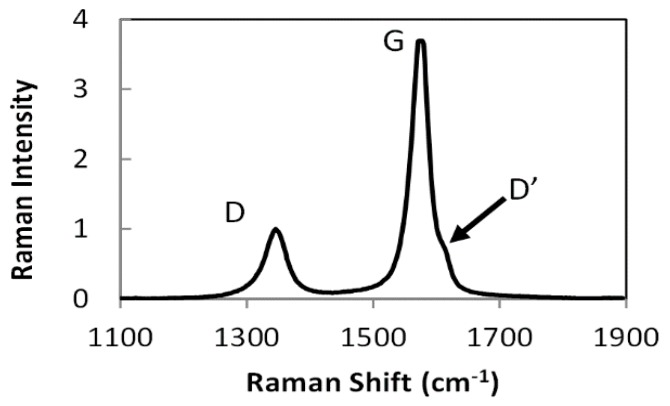
Raman spectrum of CNT sheet with G, D and D’ peaks.

In Figure 1a, the surface of a CNT sheet is shown to exemplify the randomly oriented structure in CNT assemblies. The material is porous, with approximately 100 to 200 nm spacing between the bundles that are 10 to 50 nm in diameter. The sheet in Figure 1b is stretched to 30% elongation in the horizontal direction. The image shows how the CNT network aligned to the applied stresses in a manner reminiscent of a stretched fishing net. In these assemblies, non-CNT material including iron catalyst nanoparticles and the amorphous carbons may be identified at higher magnification, as shown in Figure 2. In Figure 2a, the aggregates of iron nanoparticles are clearly captured by TEM, which are distributed unevenly on the surface of CNT bundles. Meanwhile, amorphous carbon wrapping on individual nanotubes can only be detected via a higher resolution image, as shown in Figure 2b. In order to evaluate the degree of crystallinity of the CNT assemblies, Raman spectroscopy was performed. The spectrum shown in Figure 3 spans 1100 cm^−1^ to 1900 cm^−1^ to encompass the G and D peaks that correspond to graphitic and non-graphitic carbon, respectively [36]. Additionally, the spectrum contains the D’ peak, which accounts for the shoulder on the right edge of the G peak. The ratio of the intensity of the G peak to the intensity of the D peak (I_G_/I_D_) is 3.7, indicating a reasonably good graphitic structure with minimal defect sites.

Surface wetting by DCPD was studied measuring the resin contact angle on the CNT sheets, which was performed at room temperature and without modification of the material. Before adding liquid, the sheet had a uniform matted finish. When the CNT surface came into contact with a 50 µL droplet of DCPD, a low angle drop (~10°) is momentarily formed, then the droplet disappears into the sheet after about 5 s. The final contact angle could not be measured, as the liquid infused too quickly into the porous structure to capture a static image. Once the DCPD absorbs, the central surface region wetted by the droplet is consolidated, thereby forming a depression in the sheet.

**Figure 4:**
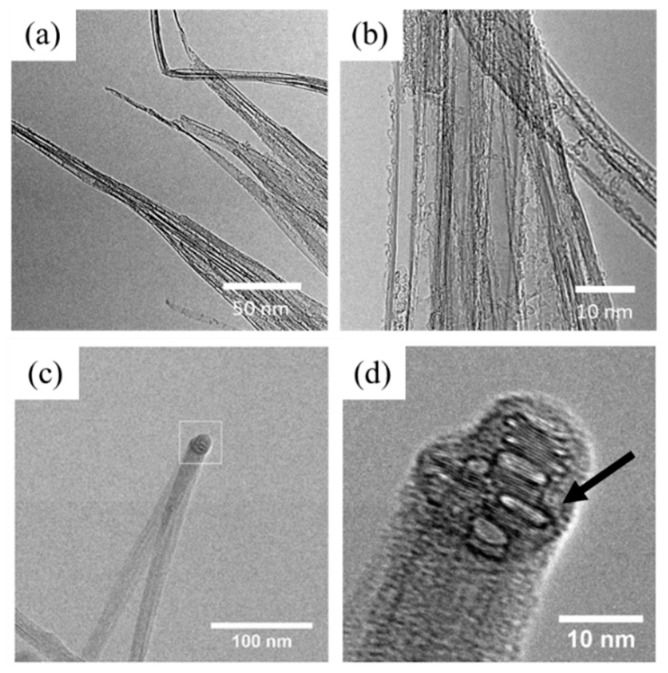
TEM investigation of the nanostructure of Dicyclopentadiene (DCPD)-infused CNT bundles. (**a**) A representative TEM image of DCPD-infused CNT bundles. (**b**) High magnification of a few bundles infused with DCPD. (**c**) A kinked DCPD–CNT bundle. (**d**) Enhanced magnification of the kink highlighted in the box in (**c**). The arrow indicates the DCPD sheath outside of the CNT bundle.

Figure 4 shows the micro- and nano-structures of DCPD-infused CNT bundles under high resolution transmission electron microscopy (HRTEM) investigation. Representative TEM images in Figure 4a,b show DCPD is thoroughly dispersed into the entire structure of CNT bundles, which straightens the individual CNTs. Interestingly, a kinked carbon nanotube bundle coated in the DCPD polymer is observed in Figure 4c,d. There are no large particles or steps on the surface, indicating a continuous film. The region indicated in the box in Figure 4c is magnified in Figure 4d, and it shows the structure of the carbon nanotubes that have been distorted by the kink, forming into oval and flattened cross-sections. The amorphous film of polymer coating the CNT bundle is highlighted with an arrow. This confirms that the thickness of DCPD films sheathing the CNTs is between 2 and 5 nm.

### 3.2. Composite Yarns

**Figure 5:**
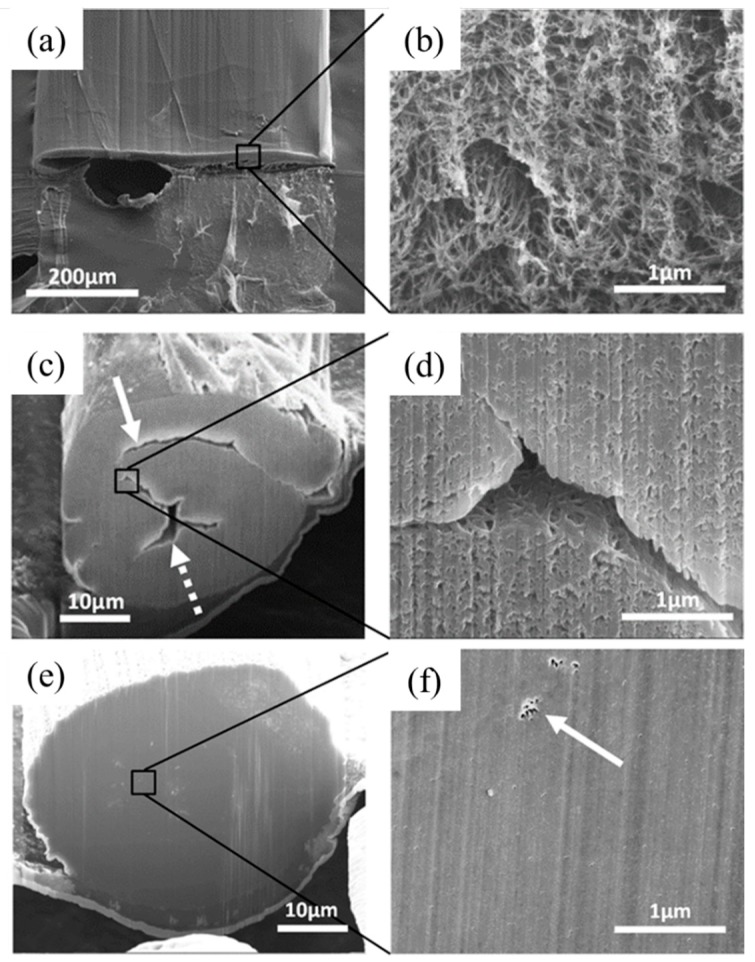
Cross-section view of scanning electron microscopy (SEM) images of different CNT yarns. (**a**) Untwisted CNT ribbon (as-received material). (**b**) High magnification revealing universal porosity. (**c**) Twisted yarn with folded structure (solid arrow) and large voids (dotted arrow). (**d**) High magnification revealing improved packing and limited micro-porosity. (**e**) DCPD-infused and consolidated CNT yarn. (**f**) Entirely solid structure with very limited nano-scale pores (solid arrow).

Figure 5 shows cross-sections of CNT assemblies in the form of as-received CNT ribbon/yarn, twisted yarn and DCPD-infused composite yarn. In Figure 5a, the pristine ribbon structure has a rectangular cross-section of approximately 20 × 500 μm. A large number of micro-pores with sizes of ~100 nm–1000 nm exist universally in the pristine yarn, as observed from the enlarged image of the cross-section in Figure 5b. After mechanical twisting, a cylindrical yarn is generated with a diameter of approximately 40 μm (Figure 5c). The solid white arrow indicates a fold where the original ribbon surface has contacted itself. The dotted arrow highlights a void with a cross-sectional area of ~800 µm^2^. Figure 5d, which has the same magnification as Figure 5b, reveals a reduced porosity within the stretched and twisted CNT yarn. Here, the pores between the nanotube bundles are on the order of 10 nm in diameter. This result suggests that mechanically twisting could effectively densify CNT assemblies that are not yet consolidated.

Moreover, a cross-section of an infused, twisted and cured composite yarn is shown in Figure 5e. It has a solid cylindrical structure with a diameter of approximately 45 μm. An increased magnification of the region boxed in Figure 5e is shown in Figure 5f, and it reveals the presence of a few isolated and nano-scaled pores, as indicated by the white arrow. Nevertheless, the rest of the surface is smooth and indistinct, because the spaces between the nanotube bundles have been filled with the polymer. In both micrographs, the faint vertical striations are an artifact of the directional ion beam used for milling.

**Figure 6:**
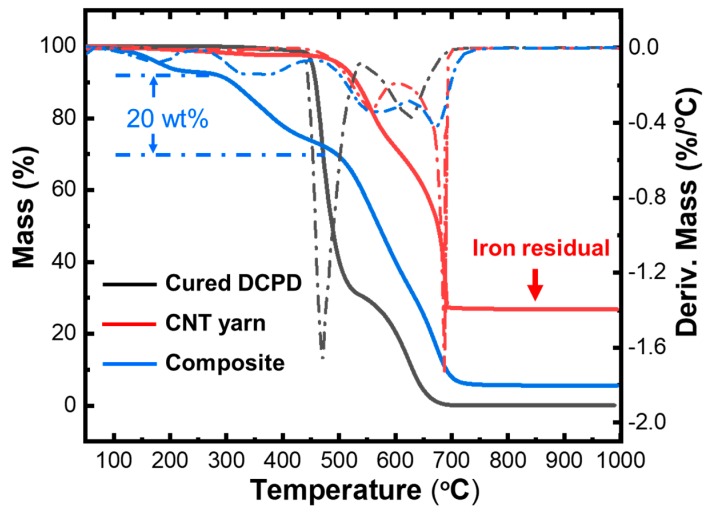
Thermogravimetric analysis (TGA) characterization of cured DCPD, CNT yarn and DCPD-CNT composite yarn. The dotted curves are derivative weights of the corresponding samples.

In order to quantify the amount of DCPD-infused in the yarn, we performed the TGA measurement of the cured DCPD, CNT yarn and composite yarn, respectively, as shown in Figure 6. The dotted curves are derivative weights of the corresponding samples, which indicate the decomposition steps of each sample. One can see that the cured DCPD has two-step decompositions with two derivative peaks at 470 °C and 626 °C (see the black dotted line). The pristine CNT yarn decomposes in two steps, too, with derivative peaks at 556 °C and 686 °C, which is likely due to the decomposition of single-walled and multi-walled CNTs, respectively (see red dotted lines). In contrast, DCPD-CNT composite yarn shows multiple-step decompositions, which has the derivative peaks at 170 °C, 376 °C, 572 °C, 674 °C. While the small initial weight loss (peak at 170 °C) can be attributed to the evaporation of moisture and to the release of some other volatiles, it is reasonable to assign the second peak (376 °C) as the DCPD decomposition in the composite. Therefore, the weight ratio of the infused resin in the composite yarn is estimated to be 20%. Moreover, we suggest the thermal stability of DCPD degrades greatly due to the intercalation of the CNT matrix, which is possibly due to the incompletely cured DCPD and the ultrathin nature of cured DCPD in the composite yarn.

**Figure 7:**
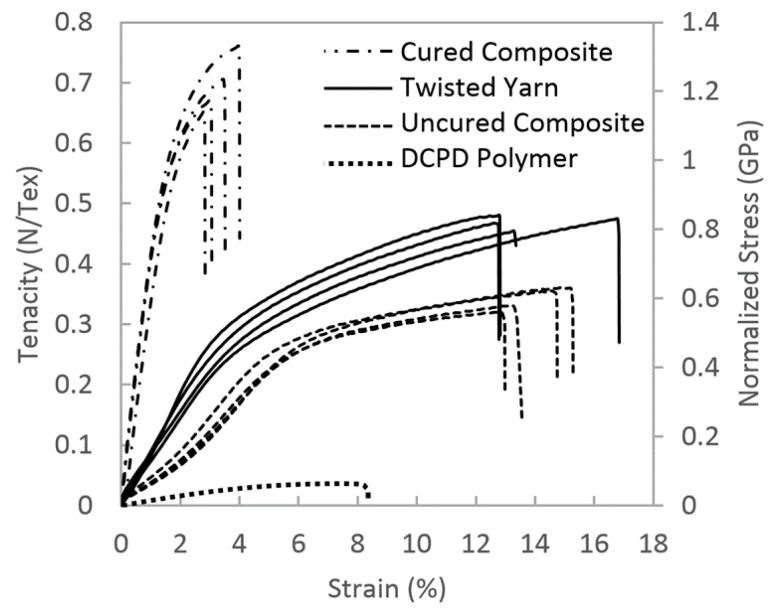
Stress–strain responses for twisted CNT yarn, cured composite yarn, uncured composite yarn and DCPD polymer.

The mechanical properties of different yarns were investigated using a tensile test. Stress–strain curves are presented in Figure 7, from top to bottom, for the cured composite yarn, the twisted yarn, the uncured composite yarn and the DCPD polymer. The mechanical properties are in N/Tex and GPa based on Equation (1) for the CNT samples. The tensile stress of DCPD is based on cross-sectional area, but it is plotted along with the CNT materials on the normalized stress scale. The modulus, tensile strength and elongation to failure values are provided in Table 1. Specifically, the polymerized DCPD had a modulus of 1.5 ± 0.1 GPa, an UTS of 0.06 ± 0.01 GPa and an elongation at failure of 8.4 ± 0.7%. The modulus, UTS and strain at failure for the twisted nanotube yarns were 12 ± 2 N/Tex, 0.4 ± 0.05 N/Tex and 14.0 ± 1.7%, respectively. The uncured composite yarns had an initial modulus of 5 ± 1 N/Tex. The strength is 0.3 ± 0.06 N/Tex, which is 25% lower than the twisted yarn, and the strain at failure, i.e., 13.8 ± 0.9%, was not significantly different. Polymerizing the DCPD in twisted CNT yarns to form a cured composite results in the Young’s modulus increasing fourfold over the twisted yarn to 55 ± 2 N/Tex. The UTS increased by 75% over the twisted yarns to 0.7 ± 0.05 N/Tex, and the elongation at failure of the composite yarn was 3.4 ± 0.4%. The stored energies up to the breaking, i.e., toughness of samples, were also calculated and summarized in Table 1. The twisted yarn and uncured composite demonstrate very high toughness, as 65.0 ± 9.8 J/m^3^ and 51.0 ± 7.0 J/m^3^, respectively. This is because they both have large strains, which are around 14%. Furthermore, compared with the pristine resin, CNT addition drastically increased the stored energy by over nine times from 2.5 ± 0.6 J/m^3^ (DCPD) to 22.9 ± 5.6 J/m^3^ (composite yarn).

**Figure 8:**
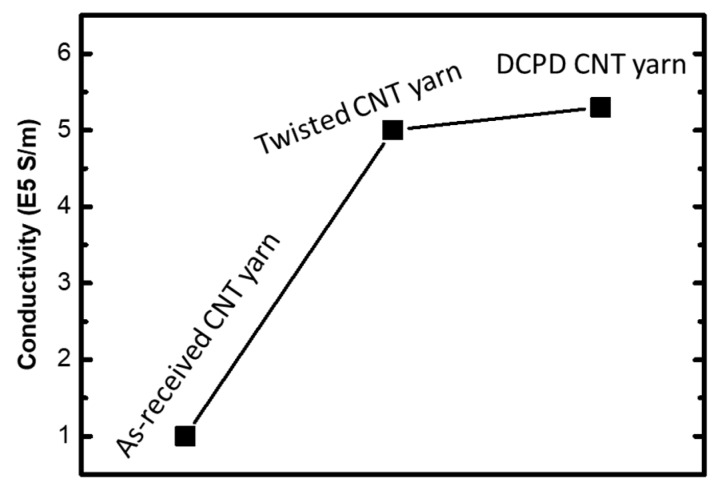
Electrical conductivity of as-received CNT yarn, twisted yarn, and DCPD–CNT yarn.

Besides the enhancement of mechanical properties, the electrical conductivity, measured using a four-probe station, increased from 1.0 × 10^5^ S/m (pristine yarn) to 5.0 × 10^5^ S/m and 5.3 × 10^5^ S/m for twisted yarn and composite yarn, respectively, as shown in Figure 8. It is not surprising to have a fivefold enhancement in the conductivity from pristine yarn to the twisted yarn, because of the consolidation that eliminated most of the porosity. The polymer-infused composite yarn presented an even higher conductivity, which is unexpected. This is likely due to a better densification brought by the polymer infusion and consolidation.

**Figure 9:**
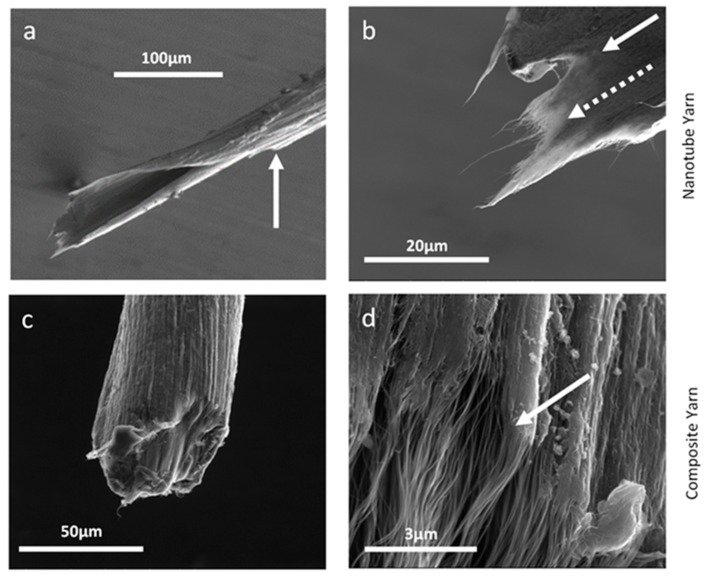
Fracture surface investigation performed by SEM. (**a**) Twisted yarn with the damage extending ~250 µm to the point indicated by the arrow. (**b**) Tip of the twisted yarn with the pull-out feature (dotted arrow) and the undisturbed structure (solid arrow). (**c**) Composite yarn with ~25 µm damage region. (**d**) Tip of the composite yarn, with arrow showing end of the resin with much smaller scale pull-out.

We further investigated fracture surfaces of a neat twisted yarn and a cured composite yarn using SEM, after breaking the sample, and the corresponding results are presented in Figure 9. The twisted CNT yarn without polymer infusion fractured over a large area. The fracture spirals back from the tip, approximately 250 μm, to the point indicated by the white arrow in Figure 9a. At higher magnification (Figure 9b), the nanotube bundles pulled out from the yarn, yielding a filamentary structure highlighted by the dotted arrow. The tip of a composite yarn (Figure 9c) showed a fairly clean fracture without cracks forming along the fiber axis. Pullout and straightening of CNT bundles from the composite yarn can be seen (solid white arrow) at a higher magnification (Figure 9d). The length of the pulled-out CNT was approximately 25 µm.

## 4. Discussion

### 4.1. Nanotube Assemblies without Resin

When CNT yarns are twisted, radial compression causes consolidation of the network. The consolidation is evident when one compares Figure 5b,d. The pore diameter in the untwisted ribbon was approximately 100 nm, versus only 10 nm in the twisted material. Folds were produced when twisting the ribbons into yarns, but the lack of bonding between the surfaces allowed them to separate. These folds are likely to reduce both the mechanical strength and the electrical conductivity of the yarn.

Without the presence of covalent bonds between nanotubes [37], or cross-linking from polymer resins, the load sharing in the yarn resulted from sliding friction between CNT bundles [38]. There are a few factors that contribute to this frictional force. The first is the presence of amorphous carbon on the CNT surface, which has been shown to increase friction when compared to the surface of pristine nanotubes [39]. Additionally, the amorphous carbon agglomerates that reside at network joints act like knots in a fishing net, and resist rearrangement of the CNT bundles. Sheet specimens were tested to understand the structural rearrangement during deformation. These served as model systems, because the structure is simplified when twists were not present. When the polymer is not present, the nanotubes are free to rearrange in response to applied forces. Friction resists the sliding apart of bundles, and amorphous carbon fixes the bundles at joints, producing a net. As a result of this net structure, the CNT yarns initially exhibit a linear stress–strain response. However, the net can only elongate so far, and eventually the only means available to accommodate increased strain is for the bundles to slide apart.

The fracture surface of the neat CNT yarn (Figure 9a) has a spiraling structure, which is a result of the twisting process. When the yarn is twisted, the core compresses radially. This causes the fibers at the core to be pushed into intimate contact, resulting in better load sharing that increases strength. The surface of the twisted yarn is not under compression, so it is the weakest point in the material, and is susceptible to crack formation. A crack will start at the weakest point and travel towards the strongest point in the progression to failure. Referencing Figure 9a, failure begins on the outer surface at the point indicated by the arrow. Then, the crack spirals to the core of the yarn, due to the helical structure between folds. The lack of bonding across the folds (Figure 5c) is likely why fracture does not occur straight across the yarn perpendicular to the load.

### 4.2. Uncured Composite

The incorporation of resin into the CNT network was carried out to bind the structure together and increase load sharing. The DCPD fully wet the CNT, as expected, due to their similar chemical composition. When infused into the network (but before polymerization), the DCPD acts as a lubricant during yarn stretching and twisting. As a result, the stress–strain curve of the uncured composite yarn is lower than that of the neat twisted yarn (Figure 7). The lubricated CNT fishing net stretches and deforms more freely, and gives rise to an initial modulus that is 57% lower than that of the twisted yarn. However, just like a fishing net that is being stretched, it can only move so far before the structure aligns and stiffens. This is the reasoning behind the increasing slope in the uncured composite yarn before yield. However, once the net has fully stretched, yield occurs, and the bundles slip apart to accommodate further deformation. Lubrication by the DCPD resin reduces the tensile stress by 26% relative to the twisted yarn. Nevertheless, the elongation at failure is the same for the uncured composite and the twisted yarn.

### 4.3. Cured Composite

The cured composite yarn, as shown in Figure 5e,f, has a solid cross section, with polymer filling all the voids between the CNT. This prevents the nanotubes from rearranging in response to stress, and distributes the load throughout the network. Within the composite yarn, the scrolled structure is still present, because the same twisting process is used for consolidation. Nevertheless, the fracture is localized, and does not propagate along the axis of the yarn. After failure, the pulled-out nanotubes remain adhered to their neighbors to form enlarged bundles, as shown in Figure 9. Tensile tests show that the cured composite yarn better utilizes the mechanical properties of the nanotubes. This effect is unique to CNT composites, as mechanical properties in carbon fiber reinforced composites are intermediate between those of the fiber and the matrix, according to the rule of mixtures [40].

### 4.4. Strengthening Mechanism

The arrangement of carbon nanotubes within the composite yarn is similar to Figure 1b, but in a composite yarn, the space between bundles is filled with polymer. This arrangement is present up to failure as the CNT rearrangement is restricted. After failure, the CNTs pullout and straighten as seen in Figure 9d. The restriction of bundle rearrangement by the polymer can account for the increased Young’s modulus of the composite yarn, but does not account for the increased tensile stress carried by the nanotubes.

**Figure 10:**
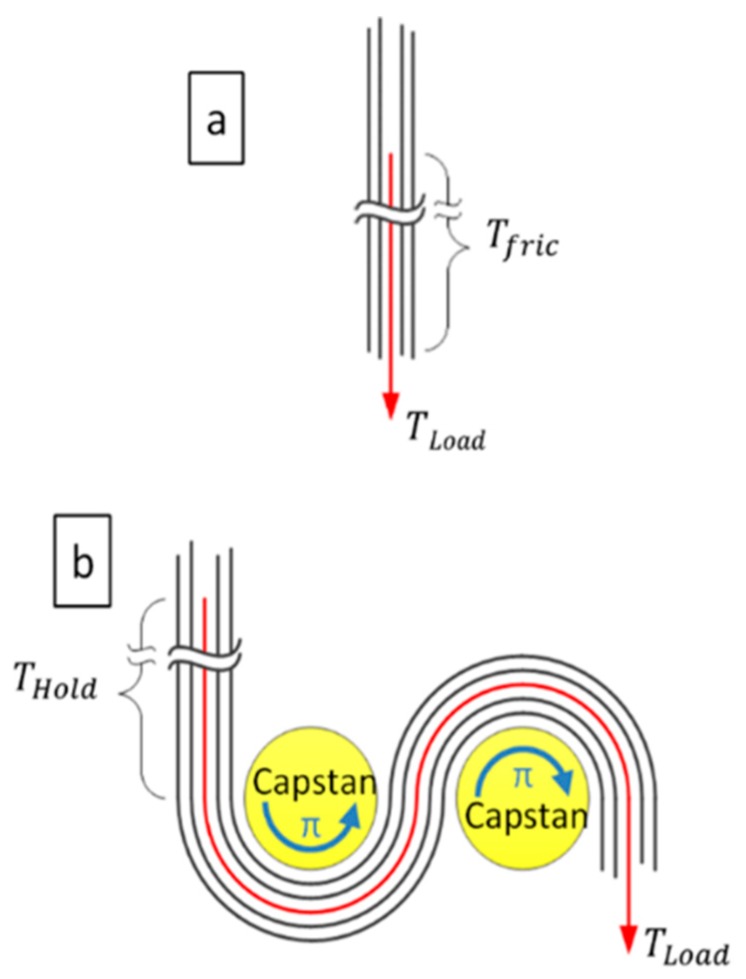
Schematic illustration of strengthening mechanism by using polymer infiltration. (**a**) Pull-out of CNT (arrow) from a pristine CNT bundle aligned by the stress. (**b**) Pull-out of CNT (arrow) when bundles curved 2π radians around DCPD capstans.

Zhang et al. [41] measured the frictional sliding force between nanotubes in a sword and sheath arrangement. They showed increased load transfer to inner shell carbon nanotubes when pulled from curved CNT sheaths. This is reminiscent of the load transfer in capstans utilized extensively for maritime applications. In a capstan, a rope is wrapped around a cylinder; there, tension tightens the rope against the cylinder inducing a normal force that increases friction [42]. Mathematically this is described by Equation (2),

(2) $$T_{Load}=T_{Hold}e^{\mu \varphi }$$

where $T_{Load}$ is the stress applied to the rope, *φ* is the angle swept around the cylinder, μ is the coefficient of friction and $T_{Hold}$ is the holding stress opposite the capstan. In Figure 10, a schematic of the capstan mechanism is given. It shows a simplified structure for how a CNT bundle could be arranged to transfer load during nanotube pullout in a twisted yarn (Figure 10a) and a composite yarn (Figure 10b). In this circumstance, CNT bundles are the rope and the DCPD polymer, if present, is the capstan.

Without cross-linked polymer filling the space between the bundles (Figure 10a), the nanotubes align with the load and eventually slip relative to each other. The CNT bundles are not curved so φ is zero, and the maximum holding stress must equal the frictional shear stress ($T_{fric}$) between the nanotubes. For the purpose of this discussion, the tensile stress inside the CNT network is estimated using Equation (1). In this case, the twisted yarns exhibit a UTS of 0.8 GPa, so considering this as $T_{Load}$ means $T_{Hold}$ could only apply 0.8 GPa to the nanotube to prevent the pullout. For the composite, case (b), the curvature is fixed in the network by filling all the pores with polymer. This polymer is assumed to be rigid for this discussion, although it would deform slightly in response to the stress. In this instance, the tortuous arrangement of CNTs is approximated as two cylinders roughly 10 nm in diameter, with the bundles in contact over 2π radians. The CNT overlap contributing to $T_{fric}$ is assumed to be 25 µm from the observed pullout in Figure 9c. The length in contact with the 10 nm capstans is insignificant when compared to the length contributing to the holding friction. Setting $T_{Hold}$ equal to the ultimate tensile stress of twisted yarns and using a coefficient of friction equal to 0.08 [43,44,45], the calculation indicates that $T_{Load}$ is 1.3 GPa. This is in reasonable agreement with our results for the composite yarns, in which the UTS is 1.2 ± 0.1 GPa obtained from the experimental measurement (Figure 7).

## 5. Conclusions

Poly(dicyclopentadiene) resin is found to fully infuse into CNT yarns forming nanocomposites with superior load transfer and improved mechanical properties. This novel resin system, with low viscosity and high CNT surface wetting, also provides lubrication during yarn processing that can be used in the future to improve microstructure and obtain higher strength. This approach also eliminates the need for employing solvents in order to reduce viscosity. In this work, the Young’s modulus and the UTS of cured composite yarns increase by 4× and 1.5×, respectively, relative to CNT yarns that have only undergone mechanical consolidation. Based on the experimental results, we proposed a capstan strengthening mechanism, which provides a new explanation for why the CNT yarns do not follow a simple rule of mixtures like traditional composite systems.

## Figures and Tables

**Table 1:** Mechanical properties of DCPD polymer, twisted yarn, uncured composite and cured composite yarn.

Sample	Modulus		UTS		Strain %	Toughness J/m^3^
	N/Tex	GPa	N/Tex	GPa		
DCPD	--	1.5 ± 0.1	--	0.06 ± 0.01	8.4 ± 0.7	2.5 ± 0.6
Twisted yarn	12 ± 2	14 ± 2	0.4 ± 0.05	0.8 ± 0.1	14.0 ± 1.7	65.0 ± 9.8
Uncured composite	5 ± 1	6 ± 1	0.3 ± 0.06	0.6 ± 0.1	13.8 ± 0.9	51.0 ± 7.0
Composite yarn	55 ± 2	68 ± 2	0.7 ± 0.05	1.2 ± 0.1	3.4 ± 0.4	22.9 ± 5.6
